# Study of Disease Severity and Outcomes in COVID-19 Patients With Chronic Kidney Disease at a Tertiary Care Hospital in South India

**DOI:** 10.7759/cureus.21413

**Published:** 2022-01-19

**Authors:** Pranav Ramamurthy, Rajashekhar R, Ashwin Kulkarni, Divya Prabhu, Anil Kumar, Rahul Ravindra, Prakriti Ramamurthy

**Affiliations:** 1 Internal Medicine, Ramaiah Medical College, Bangalore, IND; 2 Nephrology, Ramaiah Medical College, Bangalore, IND; 3 General Medicine, Ramaiah Medical College, Bangalore, IND

**Keywords:** ct severity, infection, covid-19 mortality, covid-19 severity, chronic kidney disease

## Abstract

Background: Coronavirus disease 2019 (COVID-19) disproportionately affects individuals with various comorbidities. Among these, chronic kidney disease (CKD) has been shown to be strongly associated with the progression to severe disease. This study aimed to assess the severity and disease outcomes in patients with COVID-19 infection and CKD.

Methods: This is a retrospective study conducted at a tertiary care hospital from July 2021 to September 2021. The case records of patients with CKD and COVID-19 were studied. They were compared with age and gender-matched controls equally. The presenting symptoms, clinical course, severity of illness, laboratory markers, need for ventilator support, and mortality outcomes were studied.

Results: In total, 40 CKD and 40 non-CKD patients with COVID-19 were included in the study. It was also observed that among the patients with CKD, more patients had fever, breathlessness, and diarrhea. The requirement for noninvasive ventilation, ventilator, and inotropes was on the higher average for patients with CKD. Overall mortality was 27.5% in the CKD group and 2.5% in the non-CKD group, which was statistically significant (p = 0.002).

Conclusions: COVID-19 patients with CKD had more severe illnesses with a requirement of ventilator support and had higher mortality than the patients without CKD. Patients with CKD are a key subset of patients with COVID-19 for whom more aggressive early treatment and stricter preventive measures may be beneficial.

## Introduction

The COVID-19 pandemic caused by severe acute respiratory syndrome (SARS) coronavirus-2 has caused a large number of infections and deaths worldwide. India has similarly witnessed several cases of infection [1]. Since the beginning of the pandemic, it has been vital to acquire more information about COVID-19 and the course of the potentially deadly infection. Despite a large number of infections and deaths globally, the factors influencing the severity and mortality of the illness are still not completely understood. In some individuals, the disease is asymptomatic or manifests as a mild illness, which passes quickly, whereas, in others, it can cause life-threatening pneumonia, respiratory failure, and multi-organ damage [2]. It is, therefore, imperative to analyze the occurrence of severe disease and higher mortality rates in various patient subsets to understand the factors that predispose people for more severe infection and mortality.

Many studies have attempted to observe the impact of comorbidities, such as diabetes mellitus and hypertension, as risk factors for COVID-19 [3]. Chronic kidney disease (CKD) has been studied as one of the important comorbidities that can adversely impact the course and outcome of COVID-19 infection [4]. The estimated global prevalence of CKD is approximately 13.4% [5]. In India, the approximate prevalence of CKD is 800 per million people, and the incidence of end-stage renal disease is 150-200 per million people [6]. Thus, it is not uncommon comorbidity, and it can lead to a significant mortality burden, especially when associated with COVID-19. This may necessitate the prompt identification and more aggressive treatment of COVID-19 patients with CKD compared to otherwise healthy patients with COVID-19. Studies have been conducted in western countries regarding patients with CKD, but literature in the Indian scenario is sparse. Therefore, this study was undertaken to assess the course, laboratory parameters, and severity of COVID-19 infection among patients with CKD compared to patients without CKD in an Indian hospital setting.

## Materials and methods

This is a retrospective, case-record analysis study conducted at a tertiary care center in South India from July 2021 to September 2021. The study was conducted after obtaining clearance from the Institutional Ethical Committee (MSRMC/EC/SP-08/05-2021). Data of 40 consecutive patients with pre-existing CKD admitted with a diagnosis of COVID-19 (confirmed with real-time reverse transcription-polymerase chain reaction [RT-PCR]) during the study period were retrieved from the medical records while ensuring confidentiality. Similarly, data of 40 consecutive patients without pre-existing CKD, who were matched to the cases with respect to age, gender, and comorbidities like diabetes mellitus and hypertension, were obtained. Pregnant women were excluded from the study as pregnancy alters the hemodynamic status of the body.

The patients were grouped into mild, moderate, and severe categories as per the existing Government of India Guidelines on COVID-19 [7]. The mild category included patients with symptoms of COVID-19 who were clinically stable and whose finger pulse oximetry revealed oxygen saturation of >95% in ambient air. The moderate category included patients with symptoms having oxygen saturation between 90% and 94% in ambient air or who had a respiratory rate of more than or equal to 24 cycles/min but less than 30 cycles/min. The severe category included patients with symptoms having oxygen saturation of <90% in ambient air or who had a respiratory rate of more than 30 cycles/min.

Clinical details such as complete history, examination, and investigations including serum hemoglobin, total leukocyte count, neutrophil-lymphocyte ratio, C-reactive protein (CRP), D-dimer, serum ferritin, serum creatinine, estimated glomerular filtration rate (eGFR), and high-resolution computed tomography scan of the chest (HRCT thorax) were noted. HRCT thorax was graded from 0 to 25 based on the scale devised by Saeed et al., which was observed to correlate well with the clinical severity of COVID-19 by Saeed et al. [8].

Sample size estimation

The sample size was calculated based on previous literature by Cai et al. [9]. The meta-analysis demonstrated the pooled odds ratio of 5.81 (3.78-8.94), which was used to calculate the sample size for the present study. We consider the power of the study as 80%, type I error 5%, and confidence interval 95%. With the G*Power software v3.1.9.2, the sample size was calculated as follows: z-tests - logistic regression; options - large sample z-test, Demidenko (2007) with VarCorr; analysis - A priori: compute required sample size, α err prob = 0.05 power, 1-β err prob = 0.80; critical z = 1.9599640, total sample size = 37.

Statistical analysis

The results of each parameter (numbers and percentages) for discrete data and average (mean + standard deviation) for continuous data are presented in tables. The proportions were compared using the chi-square test of significance. The student t-test was used to determine whether there was a statistical difference between male and female subjects in the parameters measured. Mann-Whitney U test was used to find out the significant difference between two independent groups in the parameters measured. In all the above tests, p-value < 0.05 was considered statistically significant. Data analysis was carried out using the Statistical Package for Social Science (SPSS, v18.5) package (IBM Corp., Armonk, NY).

## Results

In total, 80 patients were included in the study. Forty patients had pre-existing CKD, and the remaining 40 did not have CKD. In both groups, 32 patients were males, and eight were females (Table 1). The mean age was 56.4 years in the CKD group and 54.4 years in the non-CKD group. Baseline characteristics and comorbidities were matched across the groups (Table 2). Among the patients with CKD, five were in stage 2, three in stage 3, five in stage 4, and 27 in stage 5 (Table 3).

**Table 1:** Age distribution in each group CKD, Chronic kidney disease.

Group	Mean	SD	P-value
CKD (N = 40)	56.4	14.82	1.00
Non-CKD (N = 40)	56.4	14.82	

**Table 2:** Baseline characteristics between patients with and without CKD CKD, Chronic kidney disease.

Characteristics	CKD group	Non-CKD group
Males	32 (80%)	32 (80%)
Females	8 (20%)	8 (20%)
Diabetes mellitus	24 (60%)	24 (60%)
Hypertension	28 (70%)	28 (70%)

**Table 3:** Stages of CKD in the study population CKD, Chronic kidney disease.

Stages of CKD				Total
Stage 2	Stage 3b	Stage 4	Stage 5	
5	3	5	27	40
12.5%	7.5%	12.5%	67.5%	100.0%

In the CKD group, 30% of the patients had mild COVID-19, 22.5% had moderate COVID-19, and 47.5% had severe COVID-19. In the non-CKD group, 32.5% had mild COVID-19, 50% had moderate COVID-19, and 17.5% had severe COVID-19. This difference was found to be statistically significant (p = 0.008; Table 4).

**Table 4:** Severity of disease in patients with and without CKD CKD, Chronic kidney disease.

Severity	CKD (N = 40)		Non-CKD (N = 40)	P-value
Mild	30.0%	32.5%		0.008
Moderate	22.5%	50.0%		
Severe	47.5%	17.5%		

In terms of symptomatic disease, fever was observed in 85% of the patients in the CKD group and 60% of those in the non-CKD group (p < 0.001). Breathlessness was observed in 72.5% of the patients in the CKD group and 42.5% of the patients in the non-CKD group, and diarrhea was observed in 20% of the patients in the CKD group and 2.5% of the patients in the non-CKD group. These results were also found to be statistically significant (Table 5). HRCT thorax mean score was 14.73/25 (SD: 7.463) for the CKD group, and 10.9/25 (SD: 5.73) for the non-CKD group (p < 0.001).

**Table 5:** Occurrence of symptoms in patients with and without CKD CKD, Chronic kidney disease.

Symptoms	CKD (N = 40)	Non-CKD (N = 40)	P-value
Fever	85.0%	60.0%	0.012
Breathlessness	72.5%	42.5%	0.007
Diarrhea	20.0%	2.5%	0.013

Regarding other laboratory markers, no association between CKD and mean total leukocyte count, differential neutrophil count, differential lymphocyte count, or neutrophil-lymphocyte ratio was observed. Mean serum ferritin showed a statistically significant increase in patients with CKD with a p-value of 0.014. However, it is possible that this might be related to the deranged iron metabolic state in these individuals. Mean serum CRP and D-dimer levels also showed an increase in patients with CKD; however, the results were not statistically significant (Table 6).

**Table 6:** Lab parameters in CKD versus non-CKD group NLR, Neutrophil-lymphocyte ratio; CRP, C-reactive protein; CKD, chronic kidney disease; CT, computed tomography; eGFR, estimated glomerular filtration rate.

	Group	Mean	SD	P-value
Hemoglobin	CKD	10.07	1.9619	<0.001
	Non-CKD	13.21	1.6568	
NLR	CKD	7.77	8.1192	0.825
	Non-CKD	7.38	7.4729	
CRP	CKD	15.00	15.1816	0.241
	Non-CKD	11.40	11.9169	
Creatinine	CKD	6.59	4.1742	<0.001
	Non-CKD	0.96	0.3068	
D-dimer	CKD	0.89	0.653	0.333
	Non-CKD	1.12	1.3624	
CT severity score	CKD	14.73	7.463	0.012
	Non-CKD	10.90	5.737	
eGFR	CKD	23.48	34.942	<0.001
	Non-CKD	85.82	25.033	
Ferritin	CKD	533.33	374.9671	0.014
	Non-CKD	331.83	339.7383	

Noninvasive ventilator support was required in 75% of the patients with CKD and 52.5% of those without CKD. This was statistically significant (p = 0.036). Further, 35% of the patients with CKD progressed to require ventilator support compared with only 10% of those without CKD. This was also a statistically significant result (p = 0.007) (Table 7).

**Table 7:** Requirement of artificial ventilation, inotropes, mortality in CKD versus non-CKD group NIV, Noninvasive ventilation; CKD, chronic kidney disease.

	CKD (N = 40)	Non-CKD (N = 40)	P-value
NIV requirement	75.0%	52.5%	0.036
Ventilator requirement	35.0%	10.0%	0.007
Death	27.5%	2.5%	0.002

Outcomes were also poor in the CKD group with a mortality of 27.5% compared with a mortality of just 2.5% in the non-CKD group. This result was also statistically significant (p = 0.002), suggesting a direct association between CKD and higher mortality in COVID-19 infection (Table 7).

## Discussion

This study compared the clinical profile, laboratory parameters, and outcomes between COVID-19 patients with and without CKD. Patients with CKD had more severe COVID-19 disease at presentation compared to the age- and gender-matched patients without CKD. They presented with breathlessness and diarrhea more frequently compared to the patients without CKD. It was observed that most of the inflammatory parameters were similar in both the CKD and non-CKD groups. Serum ferritin was found to be significantly higher in the CKD group than that in the non-CKD group. Patients with CKD had significantly higher chest CT severity scores on average and had more severe adverse outcomes. The requirement for ventilator support and ionotropic support was higher among the patients with CKD. Similarly, the rate of mortality was higher among the patients with CKD.

Patients with CKD have an increased risk of serious infection, mainly due to an altered and reduced immune response, chronic inflammation, elevated oxidative stress, uremia, and endothelial dysfunction. Impairment of the normal reaction of the innate and adaptive immune systems in CKD predisposes patients to an increased risk of infections, virus-associated cancers, and a diminished vaccine response [10]. CKD frequently coexists with comorbidities, especially diabetes and cardiovascular disease, which are also known to be associated with worse outcomes in patients with COVID-19. CKD prevalence increases with age, and the burden of COVID-19 morbidity and mortality is heavily concentrated in older age groups [11].

Ortiz et al. have suggested that CKD is one of the important comorbidities and can be a risk factor for severe COVID-19 illness. They have also posited that patients with CKD tend to visit hospitals more frequently, and the diagnosis can be missed if the estimated glomerular filtration rate is not measured routinely [4]. This study demonstrated that patients with CKD require more ventilator and inotropic support and have higher mortality than patients without CKD. Similar results were seen in a rapid eye movement (REM) analysis, which showed that patients with CKD and COVID-19 have a higher mortality rate (pooled OR 5.81, p < 0.00001) [9]. 

The results of this study are concordant with those of Williamson et al., which demonstrated that the mortality rate is significantly higher in COVID-19 patients with CKD. The severity and mortality increase as the eGFR decreases [12]. In the present study, it was noticed that patients suffering from CKD presented with fever, breathlessness, and diarrhea. This was similar to a study by Collado et al., which showed that the presenting complaints among the patients with CKD were fever, breathlessness, and cough. The inflammatory parameters were also similar among patients with CKD [13].

Gansevoort et al. have explained that patients with CKD having COVID-19 infection need more attention and care as they are at a higher risk of severe infection and mortality [14]. Gibertoni et al. have demonstrated that the incidence of COVID-19 in patients with CKD was 4.09%, whereas it was only 0.46% in the general population. The crude mortality rate among patients with CKD and COVID-19 was 44.6%, whereas it was 4.7% in patients with CKD but without COVID-19 [15].

In a meta-analysis involving 42 studies conducted by Wang et al. on patients with COVID-19, the co-occurrence of CKD/acute kidney injury (AKI) was associated with worse outcomes compared with those without CKD/AKI [16]. As per the study by Ozturk et al., hospitalized patients with COVID-19 and CKD, including stages 3-5 CKDs, have significantly higher mortality rates than patients without kidney diseases [17].

Similarly, in the study by Abrishami et al., hospitalized patients with COVID-19 and CKDs, including stages 3-5 CKD, hemodialysis (HD), and renal transplant (RT), have significantly higher mortality rates than patients without kidney diseases [18].

In the study by Xu et al., patients with COVID-19 and CKD were older, and hypertension was the most common comorbidity. Cough and fever were present in the majority of the cases. Lymphopenia, increased D-dimer, hypersensitive C-reactive protein (hsCRP), and interleukin-6 (IL-6) were elevated in them. Ground-glass opacity and consolidation were observed in the CT scans [19].

Altogether, our results, from an Indian context, are consistent with those in the worldwide literature, showing CKD as a comorbidity for COVID-19. The limitation of the study is that the sample size was relatively small; therefore, stratified analysis of the study population could not be performed.

## Conclusions

Our study shows that CKD is a key risk factor for the severity of COVID-19. We found a positive correlation of CKD with higher CT severity scores as well as higher rates of symptomatic and severe diseases. The rate of mortality was significantly higher in patients with CKD and independent of other comorbidities. This suggests that patients with CKD are a critical subset of patients with COVID-19 who would benefit from more aggressive preventive measures, such as earlier and booster vaccination. They may also benefit from more aggressive management, even in milder cases.
